# A Case of Doxycycline-Induced Esophagitis Accompanied by Oral Aphthous Ulcers and Laryngitis

**DOI:** 10.1590/0037-8682-0454-2024

**Published:** 2025-05-09

**Authors:** Mehmet Ali Tüz, Derya Tuna Ecer, Gülce Eylül Aldemir, Muhammet Öksüzoğlu

**Affiliations:** 1 Balikesir University, Faculty of Medicine, Infectious Diseases and Clinical Microbiology Department, Balikesir, Turkey.

**FIGURE 1:**
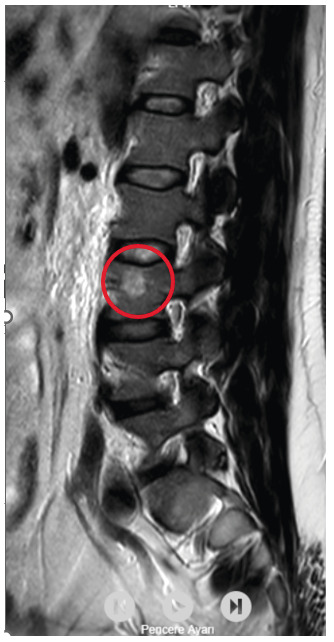
Sagittal T2-weighted images with fat suppression (T2 AG) show hyperintense signal intensity, indicating spondylodiscitis.

A 33-year-old male livestock farmer was admitted to our hospital with high fever, night sweats, and back pain. Laboratory investigations revealed a white blood cell count of 7,500/uL, a C-reactive protein level of 68 mg/L, and an 80 mm/h erythrocyte sedimentation rate. Brucella Rose Bengal and Brucella Wright (1:320 titer) tests were positive. The lumbar magnetic resonance imaging findings were consistent with those of spondylodiscitis (Figure 1). Treatment with gentamicin, rifampicin, and doxycycline was initiated based on the diagnosis of Brucella spondylodiscitis. Gentamicin was discontinued after 1 week, and the patient was discharged on an outpatient basis.

**FIGURE 2:**
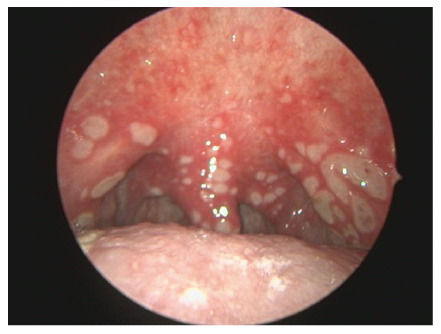
Multiple eroded white aphthous ulcers on the soft palate and uvula.

**FIGURE 3:**
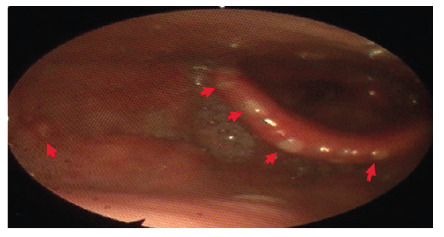
Aphthous ulcerated lesions in the larynx and epiglottis.

On the fourth day of outpatient therapy, the patient returned with sore throat and dysphagia. Physical examination revealed multiple eroded white aphthous ulcers on the soft palate and uvula (Figure 2). Subsequent laryngoscopy and endoscopy revealed similar ulcerative lesions in the larynx and esophagus (Figure 3). The medication history showed that the patient was taking doxycycline in the supine position.

Post-discontinuation of the antimicrobial therapy, the patient was prescribed an oral lidocaine mouthwash along with instructions to drink ample fluids. After 1 week, the patient’s symptoms improved with lesion regression. The patient was advised to sit upright for ≥2 hours and drink plenty of fluids after taking doxycycline. The same oral treatment regimen was restarted. The patient completed the 12-week treatment course without further symptom recurrence.

Doxycycline-induced esophagitis is a well-documented adverse event^1^. Most cases are associated with taking the drug while lying down or inadequate fluid intake^2^.
